# Controversial: Early Innate Responses to Hepatitis B Virus Infection, an Explanation for Viral Persistence?

**DOI:** 10.1007/s12250-020-00235-0

**Published:** 2020-07-06

**Authors:** Ruth Broering, Xufeng Luo, Jia Liu, Mengji Lu

**Affiliations:** 1 Department of Gastroenterology and Hepatology, University Hospital Essen, University of Duisburg-Essen, 45147 Essen, Germany; 2 Department of Infectious Diseases, Union Hospital, Tongji Medical College, Huazhong University of Science and Technology, Wuhan, 430022 China; 3 Institute of Virology, University Hospital Essen, University of Duisburg-Essen, 45147 Essen, Germany

Hepatitis B virus (HBV) infection is one of the most common causes for liver related morbidity and mortality worldwide. Liver fibrosis and cirrhosis occur in the course of chronic HBV infection, with the increasing risk to develop hepatocellular carcinoma. In about 95% of adults’ acute hepatitis B virus infection is self-limited, whereas in 90% of young children HBV infection leads to chronic progression. It is likely that a fully developed immune system contributes to HBV clearance (Dandri and Locarnini 2012; Bertoletti and Kennedy 2015). However, investigations of acute HBV infection in chimpanzees revealed a lack of any inducible immune genes related to viral entry and replication at early time points (Wieland *et al.* 2004). It has been suggested that HBV may not be recognized by the immune system and therefore has been described as stealth virus. However, the viral clearance phase is accompanied by the expression of genes related to T lymphocyte and interferon (IFN)-gamma responses. Assuming, that HBV-specific CD8 T cell effector functions and an appropriate CD4 T cell response can lead to viral clearance (Wieland and Chisari 2005). The stealth properties of HBV have recently been questioned, and it has been shown that in the early phase of infection primary human hepatocytes (PHH) sense HBV particles via toll-like receptor 2 (TLR2) (Zhang *et al.* 2020). This leads to the production of inflammatory cytokines including interleukin 6 (IL6) and tumour necrosis factor (TNF) which are able to suppress HBV replication in an interferon-independent manner (Hosel *et al.* 2009; Zhang *et al.* 2012). TLR2 activation has also been described for Kupffer cells/macrophages exposed to HBV particles *in vitro* (Cheng *et al.* 2017; Song *et al.* 2019; Zhang *et al.* 2020). This hepatic innate response results in cytokine and chemokine secretion that on the one hand may limit HBV replication and on the other hand orchestrates adaptive immune responses in the early phase of infection.

**Fig. 1:**
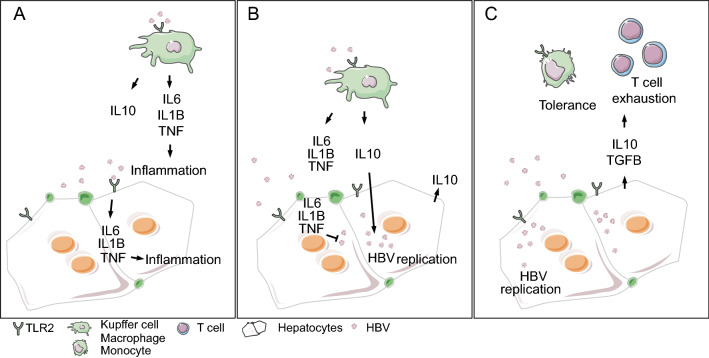
Schematic overview indicating innate immune responses in the early phase of HBV infection. The anti-inflammatory microenvironment facilitates HBV replication as well as tolerance induction and T cell exhaustion. TLR2 signaling in the early phase of HBV infection leads to production of pro-inflammatory cytokines (**A**), which limits HBV replication (**B**). However, IL10 is induced as well, subverting the anti-HBV effect (**C**).

“Stealth” virus may be not a correct description of HBV, as HBV infection induces relevant cellular responses in the early phase and the consequences of these events remain to be elusive. For example, IL10 expression may contribute to the establishment of intrahepatic immune tolerance. Activation of TLR2 signaling may delay, but do not prevent, HBV spread and persistent replication in hepatocytes (Zhang *et al.* 2020). The liver is the site of tolerance induction, and innate immune responses in TLR-stimulated PHH include pro-inflammatory cytokines (IL6, IL1B, TNF) as well as anti-inflammatory cytokines (IL10) (Broering *et al.* 2014). Repetitive stimulation of pattern recognition receptors results in faded effector functions and an IL10-mediated anti-inflammatory microenvironment. It might be suggested here, that during 4–12 weeks incubation after HBV infection, hepatic innate immune signatures facilitate the viral spread within the liver, which is characterized by an absence of viral antigens (HBeAg or HBsAg) in the blood. Interestingly, a study about acute HBV infection in a human cohort, identified a peak of IL10 serum level within the viremia phase of infection (Dunn *et al.* 2009), suggesting that once hepatic tolerance prevailed, HBV viremic phase starts, followed by symptomatic disease progression. Within this study, two asymptomatic patients only had very low IL10 levels and directly cleared the virus in the incubation period (Dunn *et al.* 2009). In murine models, Pam3Cys- and HBV-mediated hepatic TLR2 signaling has been linked with Kupffer cell expansion and KC-related IL10 production, resulting in T cell tolerance (Liu *et al.* 2018) and exhaustion (Li *et al.* 2015). Here, KCs produce IL10 upon TLR2 activation in response to hepatitis B antigens, and the elevated IL10 inhibits CD8 T cell function in HBV carrier mice. Interestingly, the presence of functional TLR2 is linked to viral persistence, whereas TLR2 deficiency results in clearance of HBV (Li *et al.* 2015). Human Kupffer cells (Cheng *et al.* 2017) and macrophages/monocytes respond to *in vitro* treatment with cell culture-derived HBV and patient-derived HBsAg, with an increase in cytokine expression as well (Song *et al.* 2019). Thus, hepatic innate immune responses are a major determinant of the progression and outcome of HBV infection (Fig. 1). Can the lack of hepatic inflammation in the early phase of infection be explained by the hepatic anti-inflammatory microenvironment? Or is the low expression of relevant TLRs or rather their refractory downstream signals due to exhaustion?

The mechanisms contributing to persistence of hepatotrophic viruses like HBV are still elusive. It is likely, that failure of HBV-specific CD8 T cell response is a major cause and is possibly mediated by the emergence of viral escape mutations, T cell exhaustion and development of regulatory T cells due to the inhibitory hepatic environment (Knolle and Thimme 2014). In addition, a shift from TH1 to TH17 CD4 T cell helps contribute to the loss of CD8 T cell effector functions. Furthermore, TLR2 expression in peripheral CD4 T cells promotes TH17 responses, which are associated with disease exacerbation in chronic HBV infection (Xu *et al.* 2017). Controversial findings have been reported in patients chronically infected with HBV (CHB). On one hand, reduced expression of TLR2 and suppressed HBsAg-dependent responsiveness to its ligand (Pam3Cys) occur in PBMC of CHB patients (Chen *et al.* 2008). On the other hand, the reduced expression of TLR2 on hepatocytes, Kupffer cells and peripheral monocytes has been described only in patients with HBeAg-positive CHB, whereas TLR2 expression significantly increases in HBeAg-negative CHB compared with healthy controls (Visvanathan *et al.* 2007). The natural course of chronic hepatitis B consists of HBeAg positive and negative phases, both of which are divided into an infection phase and a hepatitis phase. HBeAg negative phases are described as immune-reactive phases with elevated levels of liver transaminases and inflammation. Liver biopsies (GSE83148) as well as peripheral blood mononuclear cells (Song *et al.* 2019), obtained from CHB patients, show elevated gene expression of pro-inflammatory (IL1B, IL6 and TNF) and more importantly anti-inflammatory IL10 in comparison to healthy controls. It is likely, that innate immune signaling by TLR2 is involved in the hepatitis phase of CHB and is controlled by IL10 in the infection phase. This suggestion is in accordance with an analysis of TLR2 responsiveness in PBMCs of a small cohort of HBeAg-positive CHB patients, indicating that TNF and IL6 are induced in the hepatitis phase, whereas IL10 dominates the response in immune tolerant patients (Huang *et al.* 2015). However, mechanistic insights into the virus strategy to utilize TLR2 signaling to persist in patients and here especially the role of IL10 remain elusive.

Innate immunity is initiated by pattern recognition receptors, which either detects extracellular pattern like bacterial or fungal cell wall compounds mediating inflammatory responses or intracellular RNA or DNA molecules with foreign fingerprints that results in antiviral interferon-based processes. During HBV infection, the lack of hepatic expression of IFN and IFN-stimulated genes (ISGs) has primed the determination of stealth or invisible virus. During the early HBV infection, transcriptome analysis failed to show a drastic change of hepatic gene expression, especially when compared to hepatitis C virus infection, which is associated with a strong IFN response (Wieland *et al.* 2004; Wieland and Chisari 2005). This observation led to the hypothesis that HBV may evade innate immunity by passively avoiding or actively suppressing IFN induction and ISG expression. This hypothesis is supported by two additional facts. The induction of IFN production and ISG expression by immune transfer or by direct application of TLR ligands in HBV transgenic mice led to significant reduction of HBV replication and viral gene expression (Guidotti *et al.* 1996; Isogawa *et al.* 2005). In these studies, IFNs were found to destabilize HBV RNA at a posttranscriptional step and required the synergistic action of TNF in some instances. On the other side, HBV proteins like polymerase and HBeAg actively interfere with IFN signaling through the prevention of nuclear translocation of cellular transcription factors (Chen *et al.* 2013; Liu *et al.* 2015). Based on these findings, it was believed that HBV infection may be inhibited by IFNs during the early phase. However, this hypothesis may be not completely correct as shown in subsequent studies. Hydrodynamic injection is an established technique to generate mice with intrahepatic HBV replication. Mice injected hydrodynamically with a plasmid pAAV-HBV1.2 show long-lasting HBV replication in the liver and serve as an immune-competent mouse model for HBV infection (Wu *et al.* 2014). The induction of IFN response by polyI:C in this model had time-dependent effects: early polyI:C application together with HBV DNA results in persistent HBV replication at a low level while polyI:C application at later time point recruits immune cells including HBV-specific CD8  T cells and leads to complete HBV clearance. Not only the time point of IFN induction but also the type of interferon determines the outcome of viral infection. Hepatic expression of IFN-α in the hydrodynamic HBV model significantly reduces HBsAg, HBeAg and HBV DNA as well as hepatic HBcAg in the serum, whereas the expression of IFN-β only has marginal effects on viral replication. Interestingly, the hepatic IFN-β expression associates with IL10 expression (Zhou *et al.* 2017). TLR3 and RIG-I ligand polyI:C is a strong inducer of IFN-β in the liver (Broering *et al.* 2014; Wu *et al.* 2014) which again leads to suggest that an IL10-primed hepatic environment supports HBV persistence. However, based on the current knowledge there is no evidence for endogenous TLR3 or RIG-I signaling in either acute or chronic HBV infection. Therapeutic use of interferons in CHB shows low response rates, and its endogenous induction via polyI:C has been suggested for therapeutic use. Depending on the tolerogenic microenvironment in the liver, this strategy needs to be carefully questioned.

Liver-chimeric mice, that have been developed to analyse HBV infection *in vivo* have revolutionized the field (Bissig *et al.* 2010; Lutgehetmann *et al.* 2012), however one major limitation of these models is the lack of a functional immune system. More recently, HBV-infection in double-humanized mice, exhibiting a human immune system and a humanized liver, has been established. Here, HBV-infected animals show human immune responses, albeit impaired liver, chronic liver inflammation and liver fibrosis. The HBV-mediated liver disease has been linked to infiltration of human macrophages with M2-like activation phenotype (Bility *et al.* 2014). Interestingly, the induction of inflammatory cytokines includes IL6 and IL10 (Bility *et al.* 2014; Dusseaux *et al.* 2017). A donor-matched engraftment of hepatocytes further increases the infiltration frequency of human monocytes and NK cells into the HBV-infected liver (Billerbeck *et al.* 2016). Thus, hepatic innate responses in the early phase of HBV infection likely limit viral replication and promote efficient HBV-specific immune responses, while possibly driving the IL10-dependent tolerance induction and thereby viral persistence.
